# Simulation Analysis on Photoelectric Conversion Characteristics of Silicon Nanowire Array Photoelectrodes

**DOI:** 10.1186/s11671-015-0985-1

**Published:** 2015-06-30

**Authors:** Yong Zhao, Jin Yu, Li-Guang Fang, Jun Zheng, Hui-Qin Wang, Ji-Ren Yuan, Shaolong Wu, Guo-An Cheng

**Affiliations:** Department of Physics, Nanchang University, Nanchang, 330031 China; College of Physics, Optoelectronics and Energy & Collaborative Innovation Center of Suzhou Nano Science and Technology, Soochow University, Suzhou, 215006 China; Laboratory of Nanomaterial and Technology, Key Laboratory and Material Modification of the Ministry of Education, College of Nuclear Science and Technology, Beijing Normal University, Beijing, 100875 China

**Keywords:** Si nanowire, Photoelectrode, Simulation, Conversion efficiency

## Abstract

Semiconductor nanowire photoelectrochemical cells have attracted extensive attention in the light-conversion field owing to the low-cost preparation, excellent optical absorption, and short distance of carrier collection. Although there are numbers of experimental investigations to improve the device performance, the understanding of the detailed process of photoelectric conversion needs to be further improved. In this work, a thorough optoelectronic simulation is employed to figure out how the nanowire diameter, doping concentration, and illumination wavelength affect the photoelectric conversion characteristics of the silicon nanowire array photoelectrodes. We find that two balances should be carefully weighted between optical absorption and photogenerated-carrier collection, along with between short-circuit photocurrent density and open-circuit voltage. For the small-diameter nanowire array photoelectrodes, the overall absorption is higher than that of the larger-diameter ones with the most contribution from the nanowires. However, the substrate shows increasing absorption with increasing illumination wavelength. Higher doping density leads to a larger open-circuit voltage; while lower doping density can guarantee a relatively higher short-circuit photocurrent. To obtain high-light-conversion-efficiency photoelectrodes, the doping density should be carefully chosen with considerations of illumination wavelength and surface recombination. Suppressing the surface recombination velocity can effectively enhance the short-circuit photocurrent (open-circuit voltage) for the lightly (heavily) doped nanowire array photoelectrodes. Our systematical results provide a theoretical guidance for the photoelectrochemical devices based on semiconductor nanostructures.

## Background

Owing to the unique intrinsic morphology (e.g., large specific surface area and length-diameter ratio) and the resultant light-harvesting capability, semiconductor nanowire arrays (SNWAs) have attracted considerable attention and exhibit bright prospects in the optoelectronic fields [1–5]. Various device configurations have been proposed and richened and can be reduced into two main categories, i.e., solid-state [6, 7] and solid-liquid junctions [8–12]. The solid-state junction is a traditional configuration widely employed for the SNWAs in the form of *p*-*n*, *p*-*i*-*n*, or Schottky junctions [13–15]. The other prototype is usually constructed by immerging the SNWAs into an electrolyte, leading to a 3-D heterojunction which can orthogonalize the directions of the incident photons and the photogenerated-carrier collection [8–12, 16–18]. There are numbers of advantages in the latter configuration, such as uncomplicated preparation process, low cost, high efficiency of carrier collection, and so on.

The present investigations of the SNWA photoelectrodes (i.e., solid-liquid junction devices) in the applications of solar cells [9–12, 16–18], photocatalytic water splitting [19], and photon detection [10] are mostly focused on experiments. Simulation/theoretical analysis is scarce but significant for understanding the photoelectric response and improving the photoelectric conversion efficiency. Recently, Foley et al. extensively simulated the performances of silicon nanowire array (SiNWA) photoelectrode through the finite element method and declared that the SiNWA photoelectrodes exhibit much larger short-circuit photocurrent density (*J*_sc_) and photoelectric conversion efficiency (*η*) than those of the film counterpart [20]. However, the light scattering and diffraction effects are not involved in their work, where the light absorption in the NWs is simplified into according to the Lambert-Beer law. Therefore, further analysis/simulation of SNWA photoelectrodes considering the realistic light effects is meaningful to reveal the veritable photoelectric conversion process.

In this work, we intensively analyze the photoelectric conversion characteristics of SiNWA photoelectrodes by way of studying the influences of NW diameters (*d*), doping concentrations (*N*_d_), surface recombination, and illumination wavelengths (*λ*). Our results show that the photoelectric conversion characteristics are strongly dependent on the above four parameters. To achieving high-*η* SiNWA photoelectrodes, (1) the *N*_d_ should be high as soon as possible if the surface recombination can be effectively suppressed, (2) the *d* should not be too small for merely maximizing the optical absorption, and (3) the absorption enhancement should be preferentially taken into account for the long-*λ* illumination.

## Methods

**Fig. 1:**
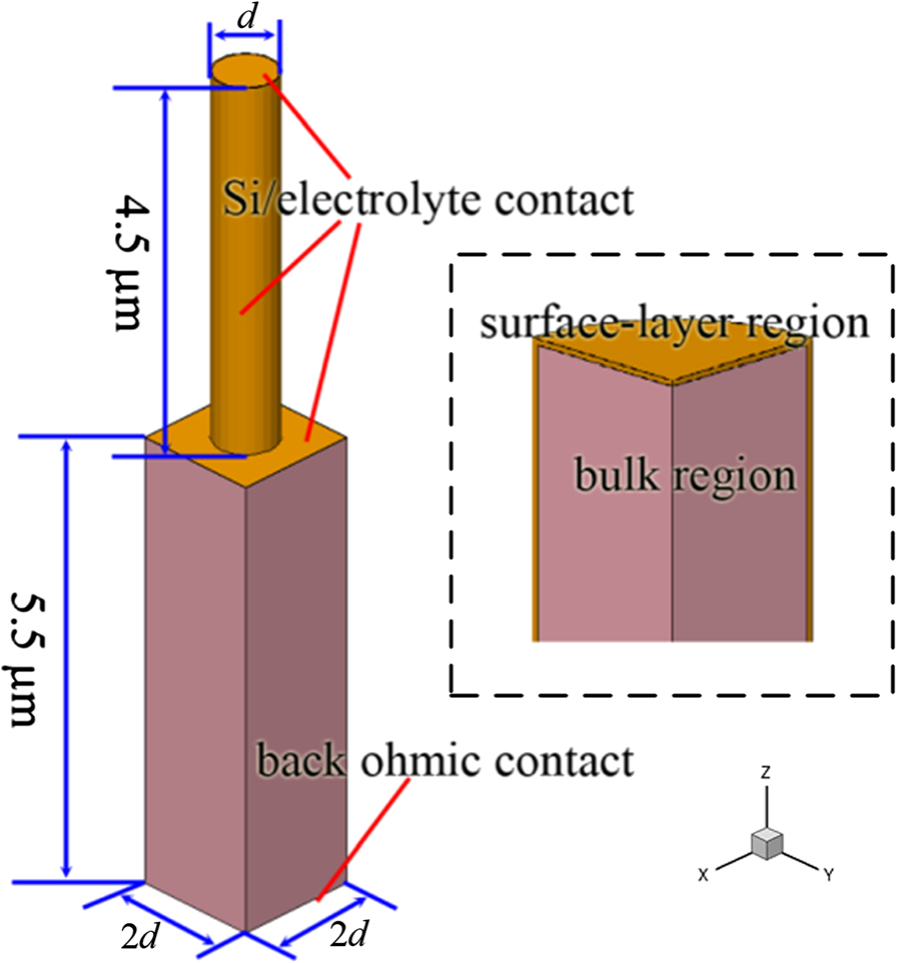
Schematic. One calculation unit of the SiNWA photoelectrode is shown with the main feature sizes. The *dashed square* indicates the details at the SiNWA/electrolyte interfaces. The incident illumination is along the *Z* direction

Uniform SiNWs are assumed to be periodic on the homogeneous substrate, i.e., the NWs are etched from the substrate. The calculation unit is shown in Fig. 1, with the diameter-to-period ratio of 0.5. In this simulation, the NW length (substrate thickness) is fixed to 4.5 (5.5) μm with consideration of the finite computation and the representative values in experiments, while the diameter varies. Optical absorption and spatial distribution of photogenerated carriers are obtained by the finite-difference time-domain method. Drift-diffusion carrier transport at 300 K is simulated by a commercial electronic design automation software (Synopsys TCAD Sentaurus Device), considering doping-dependent carrier mobility and minority-carrier lifetime, and Auger, Shockley-Read-Hall (SRH), and surface recombinations. Optical absorption and photovoltaic characteristics of SiNWA photoelectrode with different *d*, *N*_d_, *λ*, and minority-carrier lifetime in the surface layer (*τ*_sur_) are investigated in sequence.

The solid-liquid heterojunction between Si and electrolyte is assumed to be a Schottky junction with an interfacial equilibrium barrier height of 1.0 eV [11, 20]. Ohmic contact is employed on the bottom of the substrate. A 3-nm-thick surface layer in the outmost region of the front side of SiNWA photoelectrode is supposed to be rough and work as surface combination region (i.e., shell layer), where the carrier recombination occurs in the process of injecting into electrolyte. The *τ*_sur_ is usually smaller than that in the bulk (core) region from the surface recombination. The minority-carrier lifetime in the bulk (*τ*_bulk_) is dependent on the *N*_d_ in the Scharfetter relation [21]:

1 $$ {\tau}_{\mathrm{bulk}}={\tau}_{\min }+\frac{\tau_{\max }-{\tau}_{\min }}{1+{\left(\frac{N_{\mathrm{d}}}{N_{\mathrm{ref}}}\right)}^{\gamma }} $$

In our model for n-type Si (default by the employed software), *τ*_min_ is 0 s, *τ*_max_ is 2.08 × 10^−6^ s, *γ* is 1, and *N*_ref_ is 3 × 10^−6^ cm^−3^. The surface recombination is approximated to be a SRH process, which is present through deep defect levels in the gap. The surface recombination velocity (SRV) in unit of cm^−2^ s^−1^ in this model can be obtained from the *τ*_sur_ via following equation:

2 $$ \mathrm{S}\mathrm{R}\mathrm{V}=\frac{t_{\mathrm{sur}}}{\tau_{\mathrm{sur}}} $$

where *t*_sur_ is the shell thickness.

## Results and Discussion

### Influence of Nanowire Diameter

The NW sizes play a determinative role in the overall light absorption and the spatial photongenerated-carrier distributions [1, 11]. For this, we firstly assess the photoelectric conversion properties of the SiNWA photoelectrodes with different *d* (i.e., 100, 150, and 250 nm) and *N*_d_. The *N*_d_ values of the phosphor-doped Si substrates are chosen as 2.19 × 10^14^, 2.34 × 10^15^, 3.07 × 10^16^, 4.0 × 10^17^, and 1.31 × 10^18^ cm^−3^, i.e., the corresponding resistivities are around 20, 2, 0.2, 0.037, and 0.02 Ωcm, respectively. In this section, the 590-nm-*λ* illumination with the power density of 41.75 W/m^2^ is employed according to our previous experiments [10–12]. The non-polarized light is implemented by averaging two polarized lights with the angles of 0° and 90°.

**Fig. 2:**
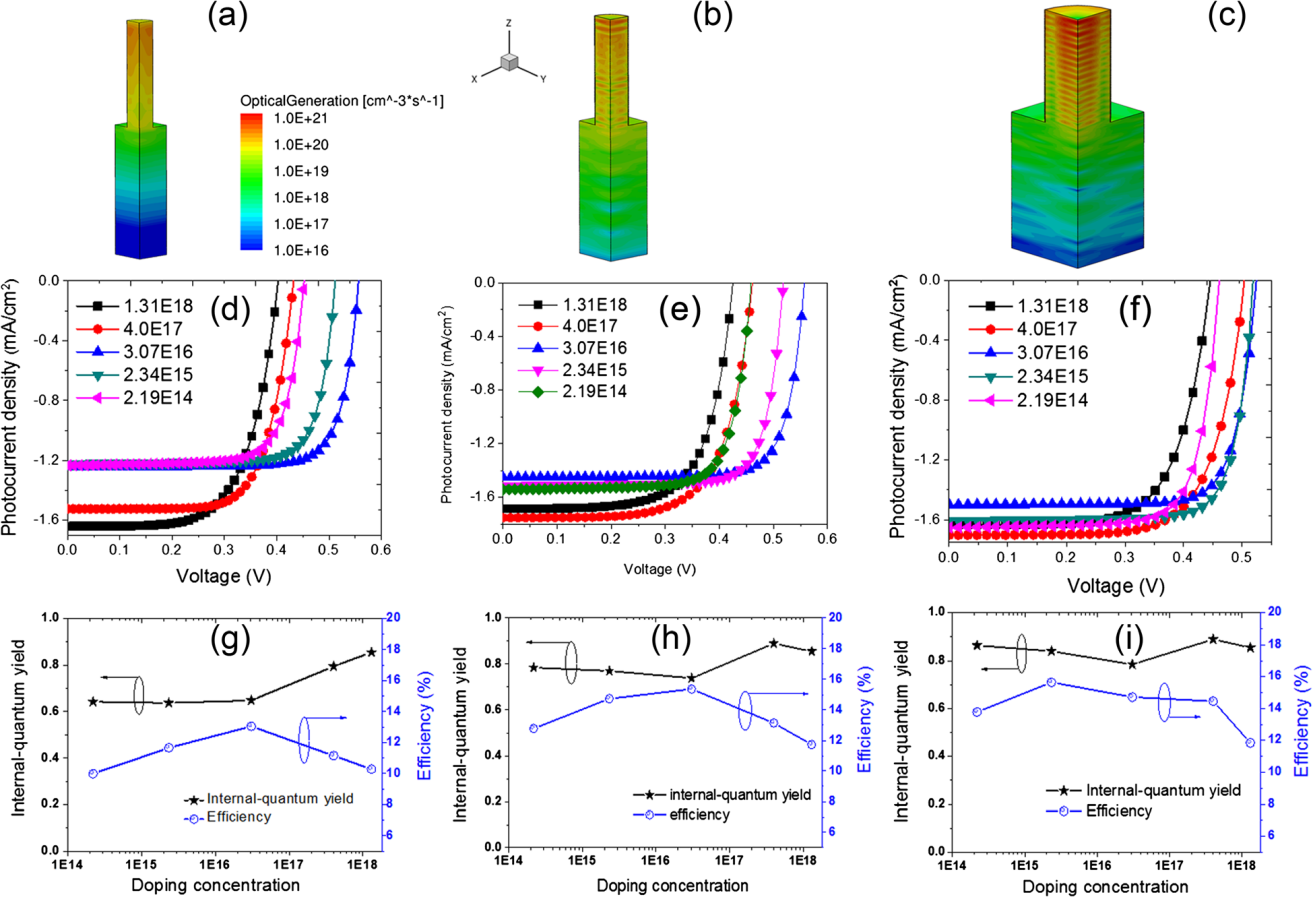
Influences of nanowire diameter. Spatial distributions of photogenerated carriers inside a quarter of the calculated unit (**a**–**c**) with *the same color bar*, *J*-*V* curves (**d**–**f**), and IQE and *η* as a function of *N*_d_ (**g**–**i**) for SiNWA photoelectrode with *d* = 100 nm (**a**, **d**, **g**), 150 nm (**b**, **e**, **h**), and 250 nm (**c**, **f**, **i**)

Figure 2a–c shows the profiles of photogenerated carriers inside a quarter of the periodical calculated unit. The overall absorption percentage (Abs) of the 100-nm-*d* SiNWA photoelectrode is 96.65 %, among of which 82.98 % is attributed to the SiNWAs (i.e., the Abs of the substrate is 13.67 %). The distributions along the NW radial direction are relatively uniform; while there are obvious attenuations along the longitudinal direction, especially inside the substrate, the carrier concentrations are close to 0 at the bottom. The Abs of the 150-nm-*d* SiNWA photoelectrode is 99.21 %, while only 66.09 % arises from SiNWAs. Inside the NWs, the distribution along the NW radial direction is not uniform, i.e., alternative peaks and valleys appear along the NW axial direction. It can be ascribed to the Fabry-Perot resonances between the substrate and the top surface of SiNWAs. The overall Abs of the 250-nm-*d* SiNWA photoelectrode reaches 96.39 % and that of the SiNWAs is up to 94.4 %. To some extent, the distributions are similar to that of the moderate-*d* SiNWA photoelectrode. Moreover, the carriers inside the substrate distribute relatively even in the NW radial direction while gradually fall off along the longitudinal direction. Thus, it can be clear that the Abs of the three photoelectrodes shows relatively small differences, but the spatial distributions of photogenerated carriers display marked differences.

Figure 2d–f plots the photocurrent density versus voltage (*J*-*V*) curves of the three photoelectrodes with different *N*_d_. Since the specific surface area of SiNWAs is quite large, the resulting surface recombination leads to *τ*_sur_ obviously smaller than *τ*_bulk_ [11]; we assume that *τ*_sur_ = *τ*_bulk_/10 in this section. For the small-*D* SiNWA photoelectrode, the *J*_sc_ gradually increases with increasing *N*_d_, and open-circuit voltage (*V*_oc_) goes up first and declines later with increasing *N*_d_. The largest *J*_sc_ (*V*_oc_) is 1.640 mA/cm^2^ (0.559 V) when *N*_d_ = 1.31 × 10^18^ cm^−3^ (3.07 × 10^16^ cm^−3^). For the moderate-*d* SiNWA photoelectrode, *J*_sc_ goes down slowly before rising up with the increase of *N*_d_ and reaches the peak (1.751 mA/cm^2^) at *N*_d_ = 4.0 × 10^17^ cm^−3^, while *J*_sc_ shows a slight decline as *N*_d_ further increases. *V*_oc_ rises first and declines later when the *N*_d_ increases from 2.19 × 10^14^ cm^−3^ to 4.0 × 10^17^ cm^−3^ and reaches the peak (0.560 V) at *N*_d_ = 4.0 × 10^17^ cm^−3^. For the large-*d* SiNWA photoelectrode, *J*_sc_ and *V*_oc_ have relatively smaller differences than the other two cases.

Internal quantum efficiency (IQE) defined by the ratio of the numbers of the carriers contributing to *J*_sc_ and the absorbed photons is employed to evaluate the collection efficiency of the photogenerated carriers. Figure 2g–i summarizes IQE and *η* for the three cases under different *N*_d_. As *D* = 100 nm, IQE goes up with the increase of *N*_d_, giving rising to the maximal IQE of 0.856 at *N*_d_ = 1.31 × 10^18^ cm^−3^. It implies that a higher *N*_d_ leads to a higher IQE for the small-*D* SiNWA photoelectrode. Meanwhile, *η* rises first and then decreases with the increase of *N*_d_, with the maximum of 13.06 % around *N*_d_ = 3.07 × 10^16^ cm^−3^. As *d* is 150 nm, IQE goes down slowly before going up, then goes down eventually. Moreover, *η* goes up before going down and reaches the maximum (15.37 %) when *N*_d_ = 3.07 × 10^16^ cm^−3^. As *D* is 250 nm, IQE has a similar change tendency with that of the 150-nm-*d* case. The maximum IQE (0.89) is achieved when *N*_d_ = 4.0 × 10^17^ cm^−3^, while *η* goes up before going down and reaches the peak (15.66 %) when *N*_d_ = 2.34 × 10^15^ cm^−3^. These results reveal that the maximal IQE and *η* cannot be simultaneously achieved for the SiNWA photoelectrodes with different *d* and *N*_d_. To get a high *η*, more factors (e.g., *V*_oc_ and fill factor) should be involved and balanced besides the IQE and *J*_sc_.

**Fig. 3:**
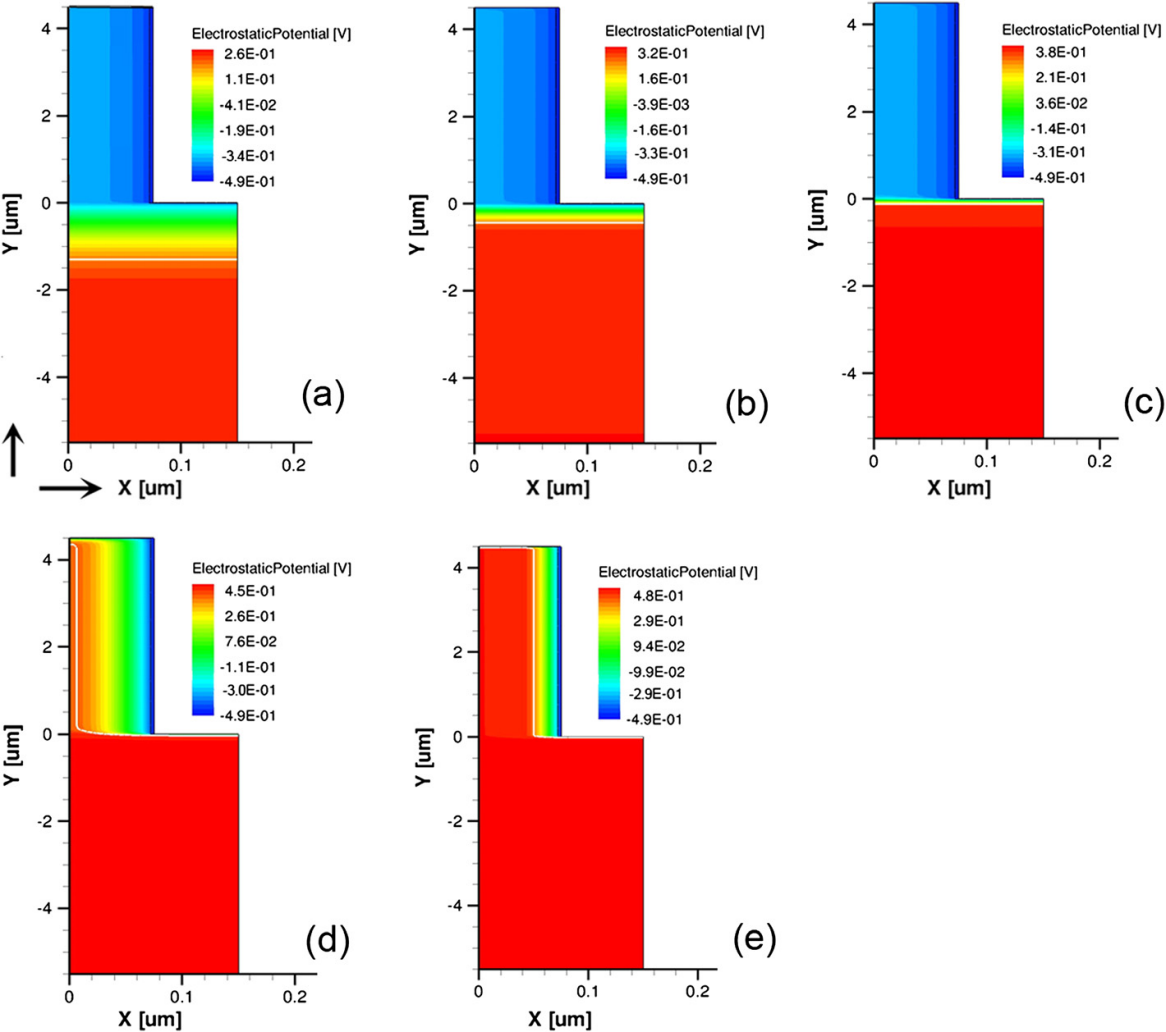
Electrostatic potential distributions. *X* axis is the NW radial direction, and *X* = 0 corresponds to the NW center; *Y* axis is the NW longitudinal direction, and *Y* = 0 corresponds to the interface between the NWs and substrate. **a**–**e** The *N*_d_ is 2.19 × 10^14^, 2.34 × 10^15^, 3.07 × 10^16^, 4.0 × 10^17^, and 1.31 × 10^18^ cm^−3^, respectively

**Fig. 4:**
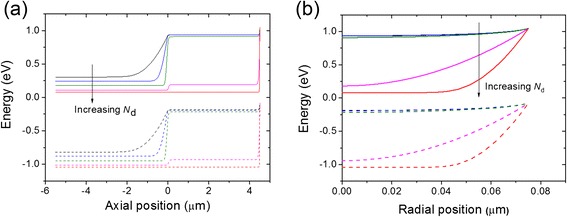
Energy band diagrams. **a** Along the NW axial direction. **b** Along the NW radial direction. *Solid* (*broken*) *lines* are the energy levels of conduction (valence) bands. Along the *arrow*, the *N*_d_ is 2.19 × 10^14^, 2.34 × 10^15^, 3.07 × 10^16^, 4.0 × 10^17^, and 1.31 × 10^18^ cm^−3^, respectively

The 150-nm-*d* SiNWA photoelectrode is taken as an example to unveil the underlying physics of the *N*_d_ influences. We dissect the profiles of electrostatic potential and energy band inside the SiNWA photoelectrode. The cross-sectional electrostatic potential profiles are plotted in Fig. 3. The depletion layer widths become thicker with decreasing *N*_d_, and leading to that, the built-in barrier between the NW-electrolyte heterojunction cannot be built completely. The energy band diagrams in the axial and radial directions are presented in Fig. 4. One can see that NWs are fully depleted as *N*_d_ < 3.07 × 10^17^ cm^−3^. The IQE of the photoelectrode sustaining high can be explained by the following: once NWs are immersed into the electrolyte, a similar *p*-*n* junction is formed at the outmost region of the substrates (as shown in Fig. 4a), which can effectively separate the photogenerated carriers in the axis direction. When *N*_d_ increases from 2.19 × 10^14^ to 3.07 × 10^16^ cm^−3^, *J*_sc_ drops slightly because of the low-efficiency collection of photogenerated carriers and the increase of SRH recombination. The increase of *V*_oc_ is mainly caused by the noticeable rise of the built-in potential in the axial direction. When *N*_d_ increases to 4.0 × 10^17^ cm^−3^, the depletion layer widths are smaller than the NW radius, leading to an obvious barrier in the radial direction (as shown in Figs 3d and 4b). In addition, the increase of *N*_d_ shortens the *τ*_bulk_ and raises *J*_0_ (i.e., exchange current density across the herterojunction interface without bias voltage), resulting in the declination of *V*_oc_ (∝1/ln*J*_0_) [20, 22]. When *N*_d_ further increases to 1.31 × 10^18^ cm^−3^, the *τ*_bulk_ is further shorten, leading to a lower *J*_sc_ than that of the case with *N*_d_ = 4.0 × 10^17^ cm^−3^ and a sustaining decrease of *V*_oc_.

### Influence of Illumination Wavelength

**Fig. 5:**
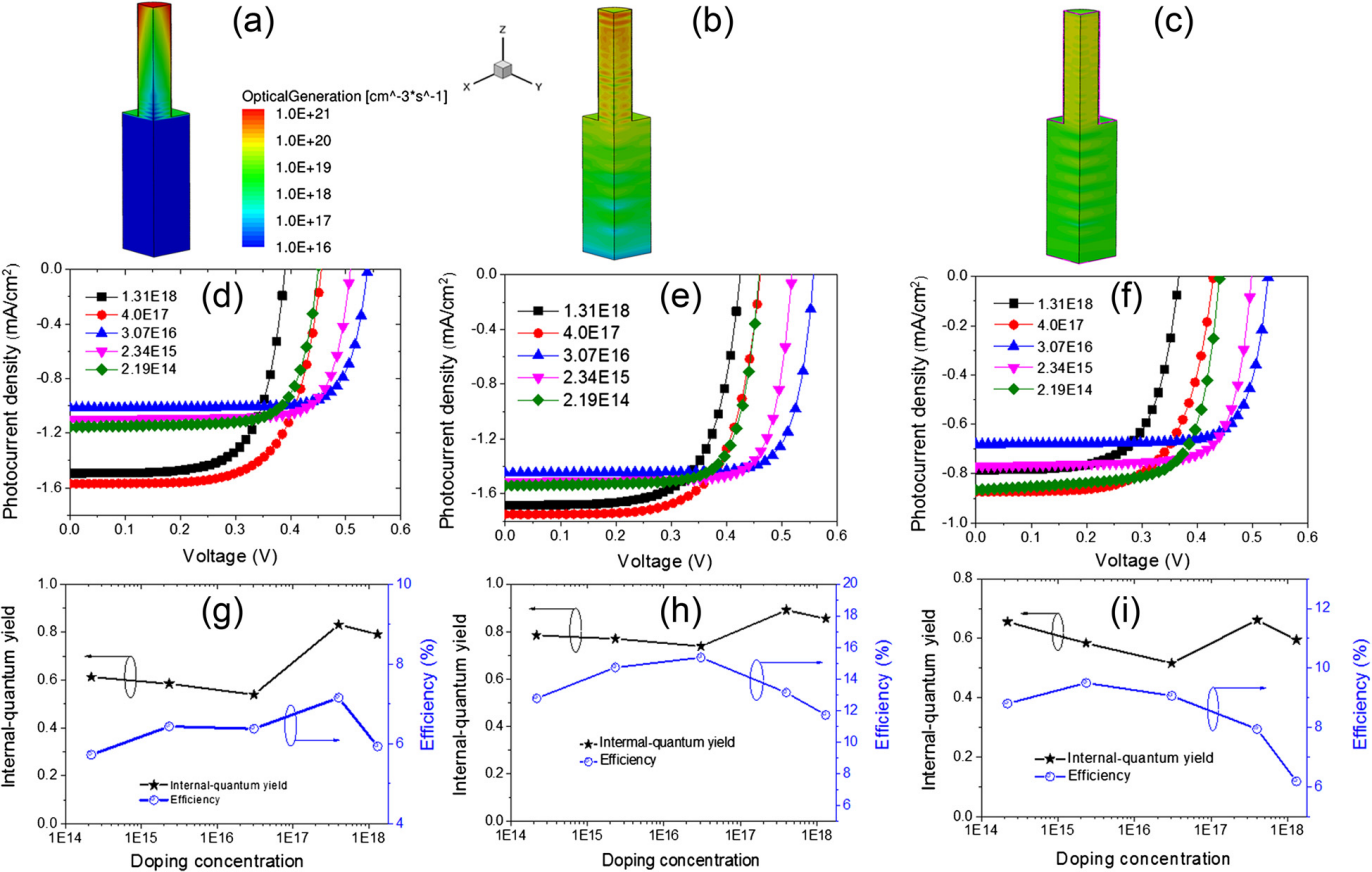
Influences of illumination wavelength. Spatial distributions of photogenerated carriers inside a quarter of the calculated unit (**a**–**c**) with *the same color bar*, *J*-*V* curves (**d**–**f**), and IQE and *η* as function of *N*_d_ (**g**–**i**) for the 150-nm-*d* SiNWA photoelectrode under the illumination of *λ* = 365 nm (**a**, **d**, **g**), 590 nm (**b**, **e**, **h**), and 800 nm (**c**, **f**, **i**). The three lights possess the same photon flux of 1.24 × 10^16^ cm^−2^ s^−1^

To fairly compare the performance and figure out the *λ* influence, we set three lights (i.e., ultraviolet (UV) light with *λ* = 365 nm, visible light with *λ* = 590 nm, and near-infrared light with *λ* = 800 nm) sharing the same photon flux of 1.24 × 10^16^ cm^−2^ s^−1^ with the corresponding power densities of 67.49, 41.75, and 30.79 W/m^2^, respectively. The spatial distributions of photogenerated carriers are presented in Fig. 5a–c. For the 365-nm-*λ* illumination, almost all of the photogenerated carriers for the 150-nm-*d* SiNWA photoelectrode are located inside the NWs and show a rapid falloff along the incident direction, which is also certified by the Abs of the entire photoelectrode (95.11 %) and the SiNWAs (95.08 %). When the illumination turns to visible (near-infrared) light, the Abs of the photoelectrode is 99.21 % (66.54 %) and that of SiNWAs is 66.09 % (35.82 %). As *λ* increases, the Abs fraction of the substrate and the entire photoelectrode increases obviously. It can be ascribed to the smaller optical absorption coefficient at longer *λ*. As a result, the photogenerated-carrier concentration inside the NWs is noticeably higher than that inside the substrate; meanwhile, the profile differences between the NWs and substrate are gradually diminished.

Figure 5d–f shows the *J*-*V* characteristics of the SiNWA photoelectrodes with various *N*_d_ under the three kinds of illumination. The *J*_sc_ and *V*_oc_ exhibit a similar variation trend with increasing *N*_d_, i.e., *J*_sc_ first decreases then increases and finally decreases (a peak around *N*_d_ = 4.0 × 10^17^ cm^−3^), *V*_oc_ first increases and then decreases (a peak around *N*_d_ = 3.07 × 10^16^ cm^−3^ ). Under the near-infrared light, *J*_sc_ is the minimum on the whole (with the highest value of only 0.873 mA/cm^2^), which can be explained by the smallest Abs. Electrostatic potential distribution in the photoelectrode is mainly determined by *N*_d_ that accounts for the week effect of illumination *λ* on the variation trends of *J*_sc_ and *V*_oc_. Figure 5g–i summarizes IQE and *η* of the SiNWA photoelectrodes with different *N*_d_ under the three lights. Since IQE is obtained from the photocurrent density divided by the incident power density, it shows the same variation trend as that of *J*_sc_. Moreover, *η* displays different variation trends with increasing *N*_d_ under the three lights. Under the UV light, *η* goes up before going down, and then again rising up before declining, giving rise to the maximum of 7.16 % when *N*_d_ = 4.0 × 10^17^ cm^−3^. Under the visible light, *η* first goes down then rises up, leading to the maximum of 15.37 % when *N*_d_ = 3.07 × 10^16^ cm^−3^. Under the near-infrared light, *η* shows the same variation tendency as that under the visible light, resulting in the maximum of 9.50 % when *N*_d_ = 2.34 × 10^15^ cm^−3^.

As a whole, *J*_sc_, IQE, and *η* under the visible light are obviously higher than those under the other two lights. Under the UV light, though *J*_sc_ and IQE are relatively large under the high-*N*_d_ conditions, *η* is low because of small *V*_oc_ and fill factor. In contrast, *J*_sc_ and *η* are always relatively small under the near-infrared light. These results imply that the NW sizes and *N*_d_, along with photoactive materials, should be re-optimized to obtain high-performance photoeletrodes under different-*λ* illuminations.

### Influence of Surface Recombination

The surface roughness and *τ*_sur_ are actually/closely related to the preparation condition of SiNWAs as well as *N*_d_. Thus, it is significant to calculate the *J*-*V* curves of the SiNWA electrodes with different *τ*_sur_ (i.e., SRV). Here, the 150-nm-*d* SiNWA photoelectrode under the 590-nm-*λ* illumination with the power density of 41.75 W/m^2^ is employed. Besides, a 400-μm-thick film photoelectrode under the same illumination is included as a comparison. The profile of photogenerated carrier in the film is produced by normal irradiation in the Beer-Lambert law, and *τ*_sur_ is supposed to equal *τ*_bulk_ for the film photoelectrode.

**Fig. 6:**
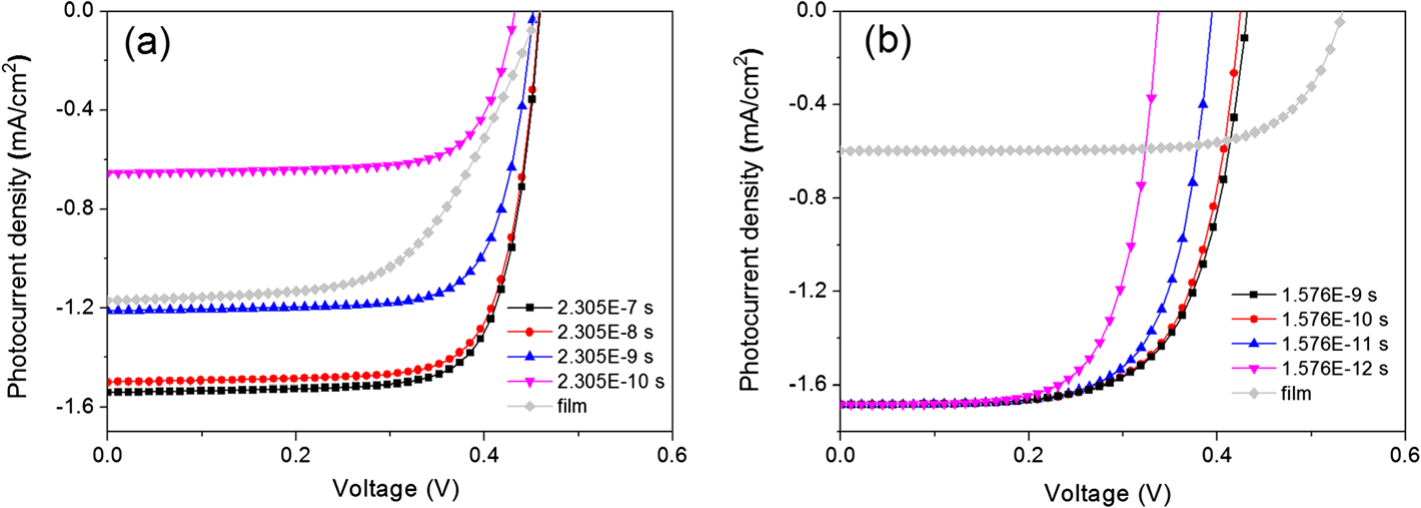
Influence of surface recombination. *J*-*V* curves of the SiNWA photoelectrodes with *N*_d_ = 2.19 × 10^14^ cm^−3^ (**a**) and 1.31 × 10^18^ cm^−3^(**b**) as the *τ*_sur_ varies. A film photoelectrode with the same *N*_d_ are shown in *gray-diamond curve* for a reference

Figure 6 plots the *J*-*V* curves of the SiNWA photoelectrodes with two presentative *N*_d_ values (i.e., 2.19 × 10^14^ and 1.31 × 10^18^ cm^−3^), with the corresponding *τ*_bulk_ of 2.035 × 10^−6^ and 1.576 × 10^−8^ s. It can be seen that the *τ*_sur_ decrease leads to a remarkable drop of *J*_sc_ for the low-*N*_d_ case. The *J*_sc_ of the SiNWA photoelectrode (1.55 mA/cm^2^) under *τ*_sur_ = *τ*_bulk_/10 is much higher than that of the film photoelectrode (1.17 mA/cm^2^). When the *τ*_sur_ reduces to *τ*_bulk_/1000, the *J*_sc_ (1.21 mA/cm^2^) is slightly higher than the film counterpart; but as *τ*_sur_ further decreases to *τ*_bulk_/10,000, the *J*_sc_ (0.65 mA/cm^2^) is substantially lower than that of the film counterpart. In contrast, the *V*_oc_ shows a slight decrease with decreasing *τ*_sur_. The *J*_sc_ variation of the SiNWA photoelectrode can be explained by the fully depleted SiNWs with low *N*_d_ and large *τ*_bulk_ (i.e., there is no radial Schottky formed in the SiNWA configuration), which brings about the collection efficiency of the photogenerated carriers in the SiNWA photoelectrode lower than that in the film photoelectrode. For the heavily doped case (i.e., *N*_d_ = 1.31 × 10^18^ cm^−3^), the *J*_sc_ slightly declines while the *V*_oc_ remarkably drops with decreasing *τ*_sur_. When *τ*_sur_ = *τ*_bulk_/10, the *J*_sc_ is 1.69 mA/cm^2^, much higher than that of the film counterpart (0.60 mA/cm^2^), while the *V*_oc_ is obviously lower. When *τ*_sur_ = *τ*_bulk_/10,000, the *J*_sc_ nearly shows no decrease; yet, the *V*_oc_ decreases from 0.53 to 0.33 V. The large margin of the *V*_oc_ falloff can be ascribed to the remarkable increase of *J*_0_. Since the NWs are partially depleted when the *N*_d_ is high, the photogenerated carrier can be rapidly separated and collected via the radial built-in electric field.

The above results reveal that the *τ*_sur_ change under different *N*_d_ has a preferential influence on different photoelectric parameters, i.e., the *J*_sc_ (*V*_oc_) of the SiNWA photoelectrode with low (high) *N*_d_ suffers great attenuation with decreasing *τ*_sur_. It suggests that the surface recombination should be suppressed to be as low as possible for the high-performance SiNWA photoelectrode in the preparation, as well as improving light absorption and Schottky barrier height.

## Conclusions

We have numerically investigated the influences of *d*, *N*_d_, *λ*, and *τ*_sur_ on the photoelectric conversion performance of the SiNWA photoelectrode. We find that (1) when the *d* is small, the overall optical absorption is up to 99 % with most contribution from the NWs, while the NWs without large *N*_d_ are totally depleted, resulting in a substantial small *J*_sc_. (2) When the *d* is large, the overall optical absorption is usually worse than that of the small-*d* one, yet the substrate contribution is larger; moreover, the NWs are partially depleted under low *N*_d_, leading to higher *J*_sc_ and *η* than those of the small-*d* SiNWA photoelectrode under the same *N*_d_. (3) Higher *J*_sc_ and IQE do not guarantee higher *η*, and photogenerated voltage and filled factor have to be involved. (4) The SiNWA photoelectrode exhibits worse light-conversion performance under the UV light than that under the near-infrared light, because under the short-*λ* illumination, most incident photons are absorbed in the near surface region, where recombination is remarkable and larger than that in the bulk. (5) An increase in the surface recombination mainly induces a great decrease in the *J*_sc_ (*V*_oc_) for the low (high)-*N*_d_ SiNWA photoelectrode. Our systematical/thorough simulation reveals the important influencing factors in the photoelectric conversion process of the SiNWA photoelectrode and gives a theoretical guidance to preparing the high-performance semiconductor nanostructure optoelectronic devices.

## Abbreviations

IQE: internal quantum efficiency
*J*-*V*: photocurrent density versus voltage
SiNWA: silicon nanowire array
SNWA: semiconductor nanowire array
SRH: Shockley-Read-Hall
SRV: surface recombination velocity
UV: ultraviolet
